# Hsp70 Promotes SUMO of HIF-1*α* and Promotes Lung Cancer Invasion and Metastasis

**DOI:** 10.1155/2021/7873085

**Published:** 2021-11-26

**Authors:** Xiean Ling, Jun Wan, Bin Peng, Jing Chen

**Affiliations:** ^1^Department of Thoracic Surgery, The Shenzhen People's Hospital, The First Affiliated Hospital of Southern University of Science and Technology, The Second Clinical Medicine College of Jinan University, Shenzhen, Guangzhou 518020, China; ^2^Emergency Department, The Shenzhen People's Hospital, The First Affiliated Hospital of Southern University of Science and Technology, The Second Clinical Medicine College of Jinan University, Shenzhen, Guangzhou 518020, China

## Abstract

**Objective:**

This study aims to investigate the effect of heat shock protein-70 (Hsp70) on epithelial-mesenchymal transition (EMT) of lung cancer cells under heat stimulation and to explore its possible molecular mechanism.

**Methods:**

qRT-PCR and immunohistochemistry assay were used to detect the expression of Hsp70 in lung cancer tissues and adjacent tissues. EdU assay was used to detect the cell activity. The effect of Hsp70 on the migration and invasion of A549 and NCI-H446 cells was detected by the wound-healing assay and Transwell assay. A tumor transplantation animal model was established to detect the effect of overexpression of Hsp70 on proliferation and metastasis of lung cancer cells. Western blot assay was used to detect the effect of thermal stimulation and overexpression of Hsp70 on SUMO modification of HIF-1*α*.

**Results:**

The wound-healing rate of A549 and NCI-H446 cells under Hsp70 stimulation was significantly higher than blank control group. At the same time, the number of cells passing through the membrane increased significantly. Hypodermic tumor transplantation in nude mice proved that knockout Hsp70 can inhibit proliferation and metastasis of lung cancer cells. Thermal stimulation upregulated the expression of Hsp70 and promoted SUMO modification of HIF-1*α*, ultimately promoting the proliferation and metastasis of lung cancer. Inhibition of Hsp70 reverses the effect of thermal stimulation on lung cancer by reducing the SUMO modification of HIF-1*α*.

**Conclusion:**

Thermal stimulation can promote EMT in A549 and NCI-H446 cells and promote cell migration and invasion in vitro and in vivo by upregulation of Hsp70. This process is associated with the promotion of SUMO modification of HIF-1*α*.

## 1. Introduction

Lung cancer is a serious threat to human health, and its morbidity and mortality rank first among all kinds of malignant tumors [1]. Despite continuous advances in surgery, chemotherapy, radiation, and biological therapy, the treatment of lung cancer, especially non-small-cell lung cancer (NSCLC), has made considerable progress [2]. However, the 5-year survival rate of NSCLC is still low due to the fact that many patients already have different degrees of metastasis at the time of visit [3]. How to detect lung cancer early and control its invasion and metastasis effectively becomes an urgent problem to be solved in lung cancer treatment [4, 5]. Epithelial-mesenchymal transition (EMT) has recently been confirmed to be closely related to the occurrence, invasion, and metastasis of tumors. EMT phenomenon also exists in lung cancer, which is related to the invasion, metastasis, and chemotherapy resistance [6–8].

Radiofrequency ablation (RFA) was found to be highly effective in early unresectable non-small-cell lung cancer (NSCLC) and metastatic lung cancer as a noninvasive treatment [9, 10]. However, thermal stimulation caused by RFA may stimulate tumor metastasis. Heat shock proteins (HSPs), also called stress proteins (sp), are a group of highly conserved proteins synthesized by human body cells under heat induction [11]. In recent years, HSPs have been found to be closely related to the occurrence, development, recurrence, and prognosis of malignant tumors. As an important member of the heat shock protein family, Hsp70 can promote the growth of tumor cells [12]. The abnormal expression of Hsp70 [13] is closely related to the occurrence and development of tumors, including tumor immunity, drug resistance, and prognosis. Nylandsted et al. [14] first found that depletion of Hsp70 would lead to death of cell lines such as breast cancer, colon cancer, prostate cancer, glioblastoma, and liver cancer. Moreover, Hsp70 expression is a prerequisite for the survival of human cancer cells. Therefore, clarifying the regulatory mechanism and active process of Hsp70 will be beneficial to elucidate the pathogenesis of tumor and provide more sufficient theoretical basis for the diagnosis and treatment of tumor [15]. However, the role of Hsp70 in lung cancer, especially whether RFA can promote tumor metastasis through Hsp70, remains to be further studied.

Hypoxia-inducible factor-1 (HIF-1*α*) is the inducible transcription factor for the heterodimer [16]. HIF-1*α* is composed of an oxygen-sensitive subunit *A* and a subunit *B* that is stably expressed in cells [17]. As a transcription factor that plays a central role in the cellular hypoxia response, HIF-1*α* regulates a variety of target genes involved in cellular adaptation and survival under hypoxia stress [18]. HIF-1*α* activation is closely associated with many pathophysiological processes, including vascular remodeling, inflammation, and ischemic hypoxic tissue damage. Small ubiquitin-related modifier (SUMO) is a member of the ubiquitin-like protein family with a molecular weight of about 12kD [19]. There are four subtypes of SUMO: SUMO1 (also known as SMT3C, Sentrin, GMP1, UBL1, and PIC-1), SUMO2 (also known as SMT3B and Sentrin-3), SUMO3 (also known as SMT3A and Sentrin-2), and SUMO4 [20]. SUMO-1 is localized in the intranuclear and nuclear pore complexes [21, 22]. SUMO-2 and SUMO-3 are expressed in nucleosomes and cytoplasm, respectively [22]. Shao et al. [21] showed that low oxygen increased the mRNA and protein levels of SUMO-1. SUMO-1 and HIF-1*α* co-localize in the nucleus under low oxygen conditions, resulting in sumoylation of HIF-1*α*. Further studies have shown that sumo-activated HIF-1*α* induces changes in its stability and transcriptional activation [20, 23].

In this study, we analyzed the expression of Hsp70 in lung cancer tissues and adjacent tissues. Furthermore, we investigated the effect of overexpression of Hsp70 on malignant progression of lung cancer. Meanwhile, the influence of thermal stimulation on Hsp70 expression was analyzed. It further detected whether Hsp70 could stabilize HIF-1*α* by promoting SUMO modification of HIF-1*α*. At present, there are few reports on the effect of Hsp70 on EMT of lung cancer cells. Therefore, this study mainly discussed the effect of Hsp70 on EMT of lung cancer cells and the related mechanism of action.

## 2. Methods

### 2.1. Clinical Sample Information and Collection

Lung cancer tissue specimens from 20 patients with complete medical history who underwent radical resection of lung cancer of our hospital from October 2018 to October 2019 were collected. At the same time, healthy control tissues of 20 lung cancer tissues (upper resection margin, 5 cm away from the edge of the tumor, >) were collected. All the tissue samples were confirmed to be lung adenocarcinoma by HE staining of routine pathological sections. The tissue was identified according to TNM staging international lung cancer TNM staging standards. The age range of the patients was 39∼76 years old. There were 13 males and 7 females. All patients signed informed consent forms. This study was approved by the Ethics Committee of Shenzhen People's Hospital.

### 2.2. Cell Culture

All cells were purchased from American Type Culture Collection (ATCC, Manassas, VA, USA). The cells were cultured in RPMI-1640 culture medium containing 10% FBS (Gibco, Life Technologies, Rockville, MD, USA) and grew at 37°C with 5% CO_2_. The medium was abandoned when the cell growth density reached 80%∼90%. After rinsing with appropriate PBS for 3 times, trypsin was added and placed at room temperature for about 2 min. Under the microscope, the cells were observed to become round and absorb the digestive juice. Complete culture medium containing 10% FBS was added to terminate digestion. Blow cells down gently and disperse them evenly according to the ratio of 1 : 3 passage. The cells were cultured in an incubator at 37°C and 5% CO_2_ until they were in the logarithmic growth stage.

### 2.3. Cell Transfection

The lung cancer cells at logarithmic growth stage were taken and the transfection kit was used according to the instructions of Lipofectamine™ 2000 (Life Technologies, Rockville, MD, USA). The cells were transfected with si-NC, si-Hsp70, vector-NC, and vector-Hsp70. The expression of Hsp70 was detected by qPCR after transfection. The transfection efficiency was verified and other experiments were performed after 48 h.

### 2.4. Immunohistochemistry

The paraffin sample was cut into 4 m thin slices. After dewaxing, the slides were placed in a 1% citric acid pressure cooker for antigen repair. One drop (50 *μ*L) of peroxidase blocking solution was added to each section to block endogenous peroxidase activity. Add 1 drop of nonimmune animal serum to each section and incubate at room temperature for 10 minutes to reduce the nonspecific background. Add 1 drop of the first antibody (Hsp70) to each section and incubate at room temperature overnight. Add 1 drop of biotin-labeled second antibody to each section. Incubate at room temperature for 10 minutes. Add 1 drop of streptomycin anti-biotin protein-peroxidase solution to each section and incubate at room temperature for 10 minutes. Each section was added with 2 drops of freshly prepared DAB (3,3-diaminobenzidine hydrochloride) (Solarbio, Beijing, China) and observed under a microscope for 2 minutes. Rinse with running water and dye with hematoxylin for 5 minutes. After dehydration, neutral resin was injected into the slices. Then, the slices were dried.

### 2.5. QRT-PCR

Total RNA was extracted from each group according to the instructions. The concentration and purity of RNA were determined and 20 *μ*L cDNA was synthesized according to the concentration of total RNA. E-cadherin forward primer 5′-CCGCCATCGCTTACA-3′, reverse primer 5′-GGCACCTGACCCTTGTA-3′. GAPDH positive primer 5′- GGATTTGGTCGTATTGGG-3′; reverse primer 5′-TCGCTCCTGGAAGATGG-3′. HIF-1*α* forward primer 5′-CTTCTGGATGCTGGTG-3′; reverse primer 5′-TCGGCTAGTTAGGGTAC-3′. Reaction conditions: two-step method, step 1: 95°C 3 min. Step 2: 40 cycles at 95°C for 10 s and 60°C for 30 s. Melting curve analysis: the fluorescence value was calculated every 5 s at a change rate from 65°C to 95°C. The following were used: relative quantitative method, determination of purpose gene, proofreading and GAPDH Ct value of PCR products, substitution formula 2^−ΔΔCt^ × 100%. The experiment was repeated three times.

### 2.6. EdU Experiment

Cells were seeded in 96-well plates with 1.0 × 10^4^ cells per well. EdU labeled cells according to the steps in the EdU imaging kit instructions (Beyotime, Shanghai, China) after incubation for 24 h. After cell fixation and infiltration promotion, EdU detection, DNA restaining, and other series of operations, the culture plates were observed under an inverted fluorescence microscope. Three fields were randomly selected to take photos. ImagePro Plus 6.0 professional image analysis software (Media Cybernetics, Silver Spring, MD, USA) was used to count the fluorescence cells to calculate the DNA synthesis rate.

### 2.7. Wound-Healing Assay

The cells were inoculated into a 6-well plate. The cells were scratched by pipette when the cell fusion rate reached 90%. The initial distance of the scratch was observed under a microscope (0 h time). After 48 hours, the distance of the scratch was measured and photographed to calculate the mobility of cells after 48 h. The experiment was repeated three times.

### 2.8. Transwell Assay

All reagents and equipment are precooled on ice. The Transwell chamber (Millipore, Billerica, MA, USA) was placed in a 24-well plate. Matrigel (Thermo Scientific, USA) 50 *μ*L (0.2 *μ*g/L) was uniformly coated on the Transwell membrane with a dilution ratio of 1 : 3. After incubation at 37°C for 15 min, the glue was solidified. After digestion, centrifugation, and counting of cells, cells were diluted with serum-free medium at 2.5 × 10^4^/ml to make the cell suspension. Cell suspension was added to the Transwell upper chamber at 200 *μ*L per well. At the same time, 10% FBS + medium 500 *μ*L was added to Transwell. Incubate at 37°C. Formaldehyde was fixed and crystal violet stained for 15 min. Then, gently wipe the cells on the lining with a cotton swab. Microscopically, the number of cells passing through the filtration membrane in four high-magnification fields (×40) was counted. The experiment was repeated three times.

### 2.9. Animal Transplantation Tumor Model In Vitro

Male nude mice aged 4 to 5 weeks were selected, weighing around 20 g. Inoculation was done 1 week after feeding. The animals were randomly divided into 2 groups with 6 animals in each group. 100 *μ*L cell suspension containing 3 × 10^6^ lung cancer cells was subcutaneously injected into the right forearm of each mouse. Tumor formation in mice was observed after 7 days. After the model was established, the size of the subcutaneous xenograft was measured with vernier caliper once every 7 days, and the observation time was 21 days. The tumor volume was calculated by the tumor volume formula: *V* = *A* × *B*^2^/2, where *V* = tumor volume, *A* = the longest diameter of tumor block, and *B* = the shortest diameter of tumor block, and the tumor growth curve of animals was plotted. At the end point, nude mice were euthanized with carbon dioxide. The nude mice were placed in the euthanasia box. Fill CO_2_ at a rate of 20% per minute replacement of the volume of the euthanasia chamber. After 10 minutes, no breathing and pupil dilation were observed in nude mice. Close the CO_2_. Observe for another 2 minutes to make sure the animal is dead. The subcutaneous tumor of the armpit was completely extirpated, and the tumor weight was weighed by the electronic balance. Animal experiments are approved by ethics committees of Shenzhen People's Hospital. All operations complied with animal welfare requirements.

### 2.10. HE Staining

Lung tissue was fixed in 10% neutral formalin solution and paraffin-embedded. 4 *μ*m slices were prepared. Xylene was dewaxed and dyed by various levels of ethanol to water and hematoxylin (Thermo Fisher Scientific, Waltham, MA, USA). Hydrochloric acid ethanol differentiation, bluing. Eosin stain, dehydrated, transparent and sealed. Fixed with neutral resin, observed under the microscope (Nikon, Japan). The tumor metastasis area was calculated by Image J software.

### 2.11. Co-Immunoprecipitation Assay

IP lysate containing proteasome inhibitor is added as required by the kit. Monoclonal antibody HIF-1*α* was added quantitatively. Incubate fully with 4°C rotary shaking. The protein-antibody complex was transferred to the centrifuge column and protein containing *A*/*G* agarose was added. Incubate in sealed rotation for 1 h. Centrifugal elution agarose. Western blot analysis was performed after boiling and denaturation of the immunoprecipitation products. The interaction between SUMO protein and HIF-1*α* protein was detected by incubation with rabbit anti-rat SUMO1 monoclonal antibody (1 : 800) and rabbit anti-rat SUMO2/3 polyclonal antibody (1 : 600).

### 2.12. Western Blot

Total cell protein was extracted. The protein concentration was determined by BCA method. SDS polyacrylamide gel electrophoresis was performed on 60 *μ*g total proteins in each group. Semidry transfer membrane, lichun red staining marks. 100 g/L skimmed milk powder was sealed at room temperature for 2 h. Diluted primary antibody, anti-Hsp70 antibody (ab2787, 1 : 1000 dilution; Abcam, Cambridge, MA, USA), anti-HIF-1 alpha antibody (ab51608, 1 : 1000 dilution), anti-Sumo 1 antibody (ab133352, 1 : 1000 dilution), anti-Sumo 2 + Sumo 3 antibody (ab81371, 1 : 1000 dilution) with 50 g/L skimmed milk powder, then incubate at 4°C overnight. TBST was used to wash 3 times at room temperature, for 10–15 min. Diluted HRP-labeled II antibody (1 : 10,000, Thermo Fisher) with skimmed milk powder of 50 mL/L and then incubate at room temperature for 2 h, and TBST was used to wash 3 times at room temperature, for 10–15 min. Detection was carried out by ECL detection system (Millipore).

### 2.13. Statistical Analysis

The experimental data were analyzed by SPSS 19.0 statistical software (SPSS Inc., Chicago, IL, USA). The experiment was repeated more than 3 times. The data were expressed as mean ± standard deviation. The comparison between the two groups was statistically analyzed by paired *t-*test. One-way ANOVA followed by Tukey's multiple comparison test was used for comparison between groups. *P* < 0.05 was considered statistically significant.

## 3. Results

### 3.1. Hsp70 Was Upregulated in Lung Cancer Patients and Lung Cancer Cell Lines

**Figure 1:**
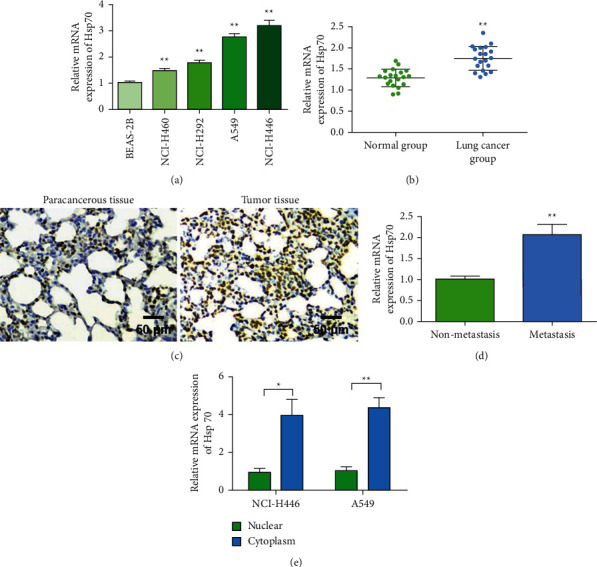
Hsp70 is upregulated in lung cancer patients and lung cancer cell lines. (a) The expression of Hsp70 is upregulated in lung cancer cell lines. (b) QPCR to detect the expression of Hsp70 in tumor tissues and adjacent tissues of lung cancer patients. (c) Immunohistochemical detection of Hsp70 expression in tumor tissues and adjacent tissues of lung cancer patients (magnification 200x). (d) The expression of Hsp70 is upregulated in patients with lung cancer metastasis. (e) qRT-PCR detects the expression and location of Hsp70 in cells. ^*∗*^*P* < 0.05, ^*∗∗*^*P* < 0.01.

To investigate the biological effects of Hsp70, we first measured the expression of Hsp70 in human normal lung epithelial cells BEAS-2B and lung cancer cells (NCI-H460, NCI-H292, A549, and NCI-H446). The experimental results showed that the expression level of Hsp70 was upregulated in lung cancer cell lines (Figure 1(a)). We found that the expression level of Hsp70 was highest in A549 and NCI-H446. Therefore, A549 and NCI-H446 were selected for subsequent experiments. QPCR was used to detect the expression of Hsp70 in tumor tissues and adjacent tissues of patients with lung cancer. The experimental results showed that the expression level of Hsp70 was increased in lung cancer tissues (Figure 1(b)). Immunohistochemistry was used to detect the expression of Hsp70 in tumor tissues and adjacent tissues of patients with lung cancer. The experimental results showed that the expression level of Hsp70 was increased in lung cancer tissues (Figure 1(c)). In addition, we also found that Hsp70 was upregulated in patients with lung cancer metastasis (Figure 1(d)). QRT-PCR was used to detect the localization of Hsp70 expression in cells. The results showed that Hsp70 was mainly expressed in the cytoplasm (Figure 1(e)).

### 3.2. Correlation Analysis of Co-Expression of Hsp70 with EMT Markers and Related Transcription Factors

**Figure 2:**
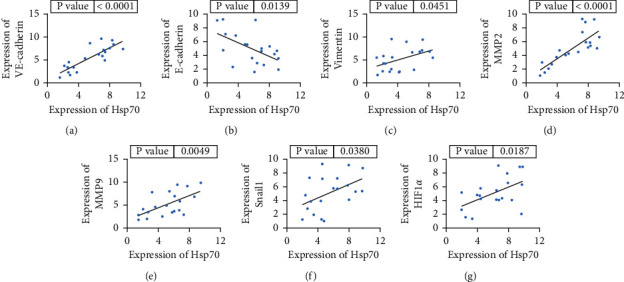
Correlation analysis of co-expression of Hsp70 with EMT markers and related transcription factors. (a) Detection of the correlation between Hsp70 and VE-cadherin co-expression. (b) Detection of the correlation between Hsp70 and E-cadherin co-expression. (c) Detection of the correlation between Hsp70 and Vimentin co-expression. (d) Detection of the correlation between Hsp70 and MMP2 co-expression. (e) Detection of the correlation between Hsp70 and MMP9 co-expression. (f) Detection of the correlation between Hsp70 and Snail1 co-expression. (g) Detection of the correlation between Hsp70 and HIF-1*α* co-expression.

Based on the collected tissues of lung cancer patients, we detected and analyzed the correlation between Hsp70 and EMT markers by qPCR. The detection results of correlation between co-expression of Hsp70 and VE-cadherin showed that there was a positive correlation between co-expression of Hsp70 and VE-cadherin (Figure 2(a)). Correlation between co-expression of Hsp70 and E-cadherin showed that Hsp70 was negatively correlated with co-expression of E-cadherin. This indicated that the expression of epithelial markers was decreased in patients with high Hsp70 expression (Figure 2(b)). Further test results showed that Hsp70 was positively correlated with Vimentin, MMP2, MMP9, Snail1, and HIF-1*α* (Figures 2(c)–2(g)).

### 3.3. Knockdown Hsp70 Inhibits the Malignant Behavior of Lung Cancer Cells

**Figure 3:**
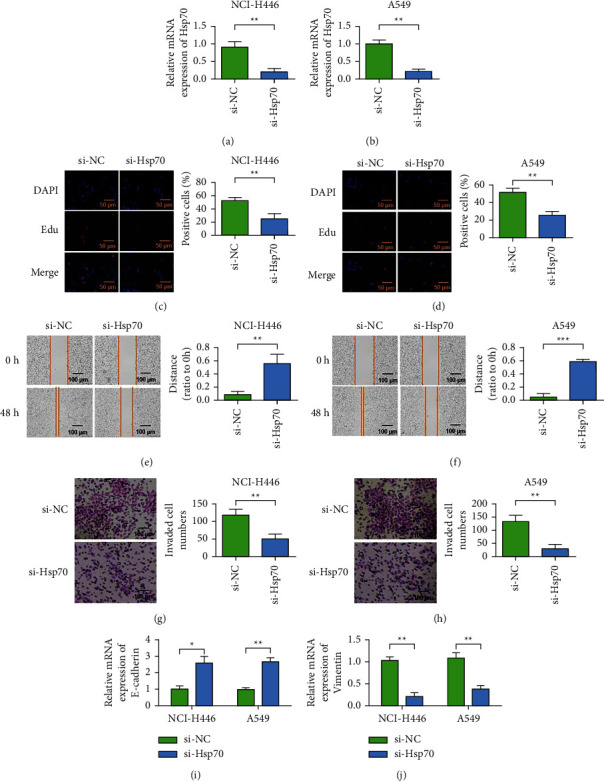
Cell level proves that knocking down Hsp70 inhibits the malignant behavior of lung cancer cells. (a) Detection of the expression level of Hsp70 in NCI-H446 cells. (b) Detection of Hsp70 expression level in A549 cells. (c) EdU detects the effect of Hsp70 on the proliferation of NCI-H446 (magnification 400x). (d) EdU detects the effect of Hsp70 on the proliferation of A549 (magnification 400x). (e) Scratch test to detect the effect of Hsp70 on the migration of NCI-H446 cells (magnification 200x). (f) Scratch test to detect the effect of Hsp70 on the migration of A549 cells (magnification 200x). (g) Transwell detects the influence of Hsp70 on the invasion ability of NCI-H446 (magnification 200x). (h) Transwell detects the influence of Hsp70 on the invasion ability of A549 (magnification 200x). (i) qRT-PCR detects the effect of knockdown of Hsp70 on the expression of E-cadherin. (j) qRT-PCR detects the effect of knockdown of Hsp70 on Vimentin expression. ^*∗*^*P* < 0.05, ^*∗∗*^*P* < 0.01.

SiRNA knockdown of Hsp70 was used to detect the effects of knockdown of Hsp70 on the proliferation, migration, and invasion of A549 and NCI-H446 cells. Detection results of Hsp70 expression showed that siRNA could effectively reduce the expression of Hsp70 (Figures 3(a) and 3(b)). Cell activity was measured by EdU assay. Cell EdU test results showed that, compared with the blank control group, cell activity decreased after Hsp70 knockdown, with a statistically significant difference (Figures 3(c) and 3(d)). Cell scratch test was performed to detect the cell migration of each group. After cell scratch treatment, serum-free culture medium was added to exclude the influence of cell proliferation on the experiment. Cell migration detection results showed that, compared with the blank control group, the scratch healing rate decreased after Hsp70 knocking, with statistically significant difference (Figures 3(e) and 3(f)). Matrigel matrix gel invasion chamber test results showed that, compared with the blank control group, the invasion ability of Hsp70 cells was decreased after Hsp70 knocking, with statistically significant difference (Figures 3(g) and 3(h)). The results indicated that knockdown Hsp70 could reduce the invasion ability of A549 and NCI-H446 cells. The results of QRT-PCR showed that after Hsp70 knockdown, E-cadherin expression intensity increased, while Vimentin expression decreased in A549 and NCI-H446 cells, with statistically significant differences (Figures 3(i) and 3(j)). The results showed that knockdown Hsp70 inhibited EMT in A549 and NCI-H446 cells.

### 3.4. Subcutaneous Tumor-Bearing Experiments in Nude Mice Proved That Knockout Hsp70 Inhibited the Proliferation and Metastasis of Lung Cancer

**Figure 4:**
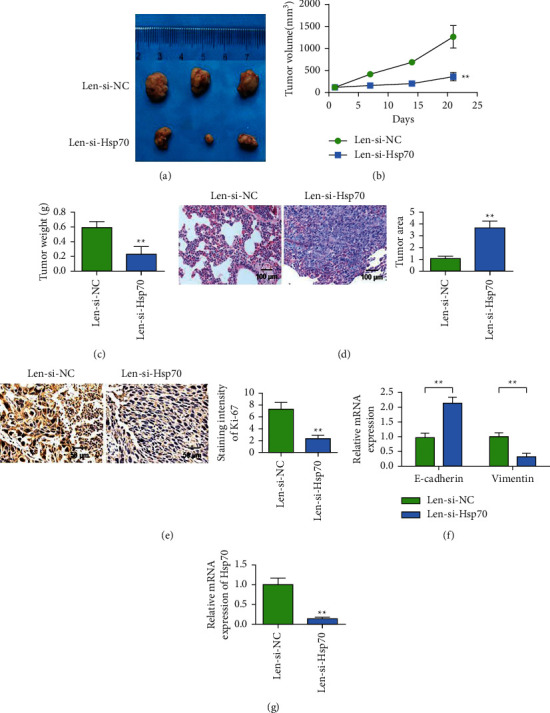
Subcutaneous tumor-bearing experiments in nude mice proved that knockdown of Hsp70 inhibits the proliferation and metastasis of lung cancer cells. (a) Pictures of nude mouse tumor. (b) Tumor growth curve of nude mice. (c) Tumor weight of nude mice. (d) HE staining of lung tissue to detect metastases (magnification 200x). (e) Ki-67 immunohistochemical staining (magnification 400x). (f) qRT-PCR detects the effect of knockdown of Hsp70 in tumor tissues on the expression of E-cadherin and Vimentin. (g) qRT-PCR detects the expression of Hsp70 in tumor tissues. ^*∗∗*^*P* < 0.01.

Subsequently, we investigated the effect of knockdown Hsp70 on proliferation and metastasis of lung cancer in a subcutaneous tumor-bearing animal model. The results of animal experiments showed that the tumor volume of Hsp70 group was smaller than that of the model control group (Figures 4(a) and 4(b)). In addition, tumor weight in the Hsp70 knockdown group was also lower than that in the model control group (Figure 4(c)). This indicated that knockdown Hsp70 could significantly reduce tumor proliferation. HE staining detected the number of tumor metastases. The results showed that knockdown Hsp70 could reduce tumor metastasis (Figure 4(d)). Immunohistochemical staining results of Ki-67 showed that the expression of Ki-67 in tumor tissues of Hsp70 knockdown group was decreased, and tumor proliferation was inhibited (Figure 4(e)). QRT-PCR results showed that in subcutaneous tumor-carrying tissues, after the Hsp70 knockdown, the expression intensity of E-cadherin in A549 and NCI-H446 cells increased, while the expression level of Vimentin decreased, and the differences were statistically significant (Figure 4(f)). The experimental results showed that compared with the control group, the expression of Hsp70 was decreased after Len-si-Hsp70 treatment (Figure 4(g)).

### 3.5. Thermal Stimulation Upregulated the Expression of Hsp70 and Promoted the Proliferation and Metastasis of Lung Cancer

**Figure 5:**
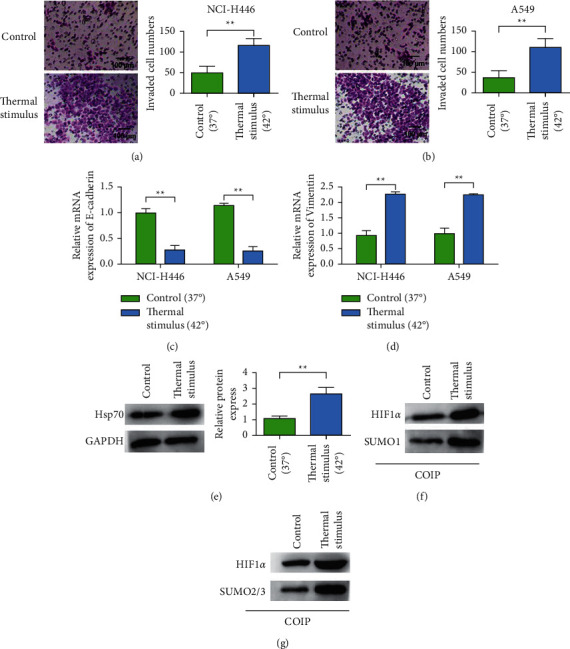
Thermal stimulation upregulates the expression of Hsp70 and promotes lung cancer proliferation and metastasis. (a) Transwell detects the influence of thermal stimulation on the invasion ability of NCI-H446 (magnification 200x). (b) Transwell detects the effect of thermal stimulation on the invasion ability of A549 (magnification 200x). (c) qRT-PCR detects the effect of thermal stimulation on the expression of E-cadherin. (d) qRT-PCR detects the effect of thermal stimulation on vimentin expression. (e) Western blot to detect the effect of thermal stimulation on the expression of Hsp70. (f) Western blot to detect the effect of thermal stimulation on the expression of SUMO1 on HIF-1*α*. (g) Western blot detects the effect of thermal stimulation on the SUMO2/3 of HIF-1*α*. ^*∗∗*^*P* < 0.01.

Transwell was used to detect the effect of thermal stimulation on the invasion capacity of NCI-H446. The experimental results showed that thermal stimulation could promote the invasion ability of NCI-H446 cells (Figure 5(a)) [24–26]. Similarly, thermal stimulation also promoted the invasion ability of A549 cells (Figure 5(b)). The results of qRT-PCR assay showed that the effect of thermal stimulation on E-cadherin and Vimentin expression showed that thermal stimulation could reduce E-cadherin expression and promote Vimentin expression in NCI-H446 and A549 cells (Figures 5(c) and 5(d)). The influence of thermal stimulation on Hsp70 expression was detected by Western blot. The results showed that thermal stimulation could upregulate the expression of Hsp70 (Figure 5(e)). Further test results showed that thermal stimulation could also upregulate the expression of HIF-1*α*, and the SUMO1-HIF-1*α* binding protein expression was significantly increased in the thermal stimulation group compared with the NC group (Figure 5(f)). Compared with the NC group, SUMO2/3-HIF-1*α* binding protein expression was also significantly increased in the heat stimulation group (Figure 5(g)).

### 3.6. Inhibition of Hsp70 Reverses the Promotion Effect of Thermal Stimulation on Lung Cancer by Reducing the SUMO Modification of HIF-1*α*

**Figure 6:**
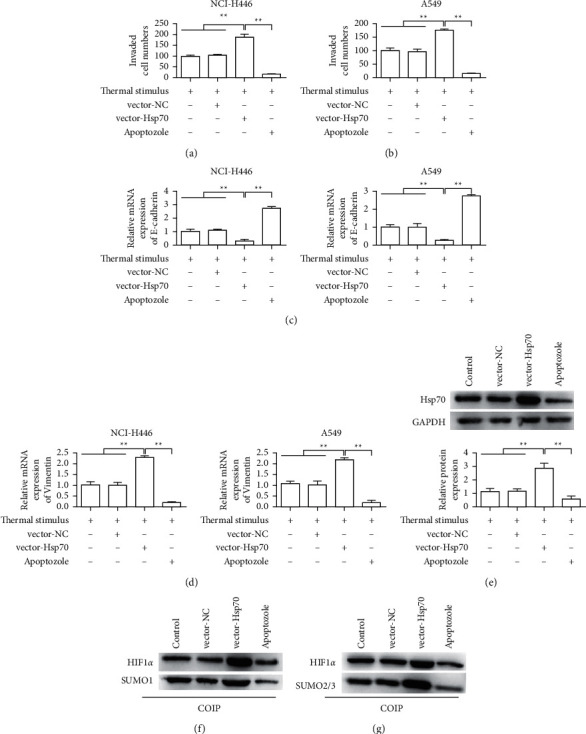
Inhibition of Hsp70 reverses the effect of thermal stimulation on lung cancer by reducing the SUMO modification of HIF-1*α*. (a) Transwell detects the influence of different treatments on the invasion ability of NCI-H446. (b) Transwell detects the influence of different treatments on the invasion ability of A549. (c) qRT-PCR detects the influence of different treatments on the expression of E-cadherin. (d) qRT-PCR detects the effect of different treatments on vimentin expression. (e) Western blot to detect the influence of different treatments on the expression of Hsp70. (f) Western blot for detection of the effects of different treatments on the expression of SUMO1 on HIF-1*α*. (g) Western blots to detect the effects of different treatments on the SUMO2/3 of HIF-1*α*. ^*∗∗*^*P* < 0.01.

The results of the transfection efficiency of the Hsp70 overexpressed plasmid were shown in Figure S1. The results showed that the overexpressed plasmid could upregulate the expression of Hsp70 compared with the control group. Transwell assay was used to detect the effect of different treatment on the invasion ability of NCI-H446 and A549 cells. The results showed that overexpression of Hsp70 promoted the invasion of NCI-H446 and A549 cells compared with the control group. The Hsp70-specific inhibitor Apoptozole can inhibit the invasion of NCI-H446 and A549 cells (Figures 6(a) and 6(b)). The results of western blot showed that overexpression of Hsp70 could inhibit the expression of E-cadherin and promote the expression of Vimentin. However, the Hsp70-specific inhibitor Apoptozole could inhibit the expression of Vimentin and promote the expression of E-cadherin (Figures 6(c) and 6(d)). The results of western blot showed that the overexpressed plasmid could upregulate the expression of Hsp70, and Apoptozole could inhibit the expression of Hsp70 (Figure 6(e)). Further detection results showed that overexpression of Hsp70 could also upregulate the expression of HIF-1*α*, and the SUMO1-HIF-1*α* binding protein expression was significantly increased in the Hsp70 group compared with the NC group (Figure 6(f)). Compared with the NC group, SUMO2/3-HIF-1*α* binding protein expression was also significantly increased in the Hsp70 overexpressed group (Figure 6(g)). Apoptozole was able to inhibit the SUMO1-HIF-1*α* and SUMO2/3-HIF-1*α* protein complexes and reduce their expression in Figures 6(f) and 6(g).

## 4. Discussion

Invasive metastasis is the leading cause of treatment failure in lung cancer [27]. Recent studies have shown that EMT plays a very important role in the distant metastasis of lung cancer and the occurrence of chemotherapy resistance [28]. Therefore, the study on EMT is helpful for the prevention and treatment of tumors [29–31].

As a molecular chaperone, Hsp70 is highly expressed in a variety of malignant tumors. Hsp70 is closely related to the proliferation, apoptosis, and prognosis of most malignant tumor cells, and plays an important role in the occurrence and development of malignant tumors. Clarifying the regulation mechanism and active process of Hsp70 will be beneficial to elucidate the pathogenesis of the tumor. It also provides a more adequate theoretical basis for the diagnosis and treatment of tumors. Normal cells expressed only a small amount of Hsp70. Hsp70 is involved in cell growth and metabolism and is strictly regulated by the cell cycle [32]. However, the tumor cells can be independent of the cell cycle and show abnormal distribution and localization of Hsp70 induced by continuous high expression even under the condition of no stress. For example, Hsp70 expression in ovarian cancer cells is several times higher than that in normal ovarian cells [33]. Beer et al. showed that overexpression of Hsp70 in early lung adenocarcinoma is a molecular marker of poor prognosis [34]. Hsp70 may also be a sensitive indicator of early hepatocellular carcinoma [35].

E-cadherin is a calcium-dependent transmembrane protein which mainly mediates adhesion between cells [36, 37]. E-cadherin is a phenotypic characteristic protein of epithelial cells, which is widely involved in intercellular connectivity. Vimentin is a kind of cytoskeleton protein, which is not expressed in normal epithelial cells, but widely distributed in various endothelial cells, fibroblasts, macrophages, lymphocytes, and other mesenchymal cells, and is an important factor of EMT [38]. Decreased E-cadherin expression is recognized as a marker of loss of epithelial cell characteristics. This study confirmed that Hsp70 can significantly inhibit the expression of E-cadherin and promote the expression of Vimentin. It suggests that the expression level of E-cadherin is related to the level of Hsp70. MMPs plays a crucial role in tumor invasion and metastasis. MMPs is a zinc ion-dependent endocrine protease, which is mainly involved in the regulation of intercellular adhesion. MMPs can degrade various protein components of extracellular matrix and promote angiogenesis [39]. The degradation of extracellular matrix helps tumor cells to break through the basal membrane and spread outward. Present study also found that Hsp70 levels in the tissues of lung cancer patients with distant metastasis were higher than those without metastasis. Correlation analysis also showed that Hsp70 was negatively correlated with the co-expression of E-cadherin. Hsp70 was positively correlated with the co-expression of Vimentin, VE-Cadherin, MMP2, MMP9, Snail1, and HIF-1*α*. In this study, it was found that after Hsp70 stimulation, the movement and invasion ability of lung cancer cells was concentration-dependent, showing another important characteristic of EMT. The related mechanism may be related to the expression changes of E-cadherin, Vimentin, and MMPs.

HIF-1 is a key transcription factor for cell adaptation to hypoxia [40]. Ubiquitination and SUMO are posttranslational modifications that can regulate their protein levels and transcriptional activation activities. It was found that overexpression of SUMO-1 significantly increased HIF-1*α* protein levels and transcriptional activation activity under anoxic conditions. Further studies confirmed that SUMO-1 sumoylation of HIF-1*α* resulted in increased stability and nuclear expression of HIF-1*α*, leading to enhanced transcriptional activation of HIF-1*α*. In this study, it was found that thermal stimulation could upregulate the expression of Hsp70, thus promoting the modification of HIF-1*α* by SUMO1 and SUMO2/3. The Hsp70-specific inhibitor Apoptozole reverses the effect of thermal stimulation on lung cancer by reducing the SUMO modification of HIF-1*α*.

## 5. Conclusion

In summary, this study confirmed in vitro that Hsp70 can induce EMT in lung cancer cells and enhance the migration and invasion ability of lung cancer cells. This process is involved in promoting SUMO modification of HIF-1*α*. Further study found that thermal stimulation can upregulate the expression of Hsp70. These results provide a new direction for further research on prevention strategies and therapeutic targets of lung cancer invasion and metastasis, and lay a theoretical foundation for animal experiments and clinical studies in the future.

## Data Availability

The data used to support the findings of this study are available from the corresponding author upon request.

## Supplementary Materials

Supplementary Materials Figure S1: transfection efficiency detection results of Hsp70 overexpressed plasmid. The results showed that the overexpressed plasmid could upregulate the expression of Hsp70 compared with the control group.
