# Supplementary material for: Hsp70 Promotes SUMO of HIF-1α and Promotes Lung Cancer Invasion and Metastasis
Source: J Oncol. 2021 Nov 26;2021:7873085. doi: 10.1155/2021/7873085 (PMC8642011; doi:10.1155/2021/7873085)
Supplement: Supplementary Materials — Figure S1: transfection efficiency detection results of Hsp70 overexpressed plasmid. The results showed that the overexpressed plasmid could upregulate the expression of Hsp70 compared with the control group. [file 7873085.f1.docx]

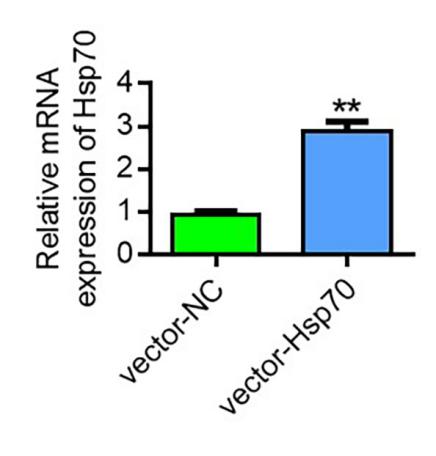


**Figure S1. The transfection efficiency detection results of Hsp70 overexpressed plasmid.** The results showed that the overexpressed plasmid could up-regulate the expression of Hsp70 compared with the control group.
